# Exerkines and osteoarthritis

**DOI:** 10.3389/fphys.2023.1302769

**Published:** 2023-12-01

**Authors:** Shuangshuo Jia, Ziyao Yu, Lunhao Bai

**Affiliations:** ^1^ Department of Orthopedic Surgery, Shengjing Hospital of China Medical University, Shenyang, China; ^2^ Imaging Department, Dalian Medical University, Dalian, China

**Keywords:** exerkine, osteoarthritis, exercise, myokine, cardiokine, adipokine

## Abstract

Osteoarthritis (OA) is the most prevalent chronic joint disease, with physical exercise being a widely endorsed strategy in its management guidelines. Exerkines, defined as cytokines secreted in response to acute and chronic exercise, function through endocrine, paracrine, and/or autocrine pathways. Various tissue-specific exerkines, encompassing exercise-induced myokines (muscle), cardiokines (heart), and adipokines (adipose tissue), have been linked to exercise therapy in OA. Exerkines are derived from these kines, but unlike them, only kines regulated by exercise can be called exerkines. Some of these exerkines serve a therapeutic role in OA, such as irisin, metrnl, lactate, secreted frizzled-related protein (SFRP), neuregulin, and adiponectin. While others may exacerbate the condition, such as IL-6, IL-7, IL-15, IL-33, myostatin, fractalkine, follistatin-like 1 (FSTL1), visfatin, activin A, migration inhibitory factor (MIF), apelin and growth differentiation factor (GDF)-15. They exerts anti-/pro-apoptosis/pyroptosis/inflammation, chondrogenic differentiation and cell senescence effect in chondrocyte, synoviocyte and mesenchymal stem cell. The modulation of adipokine effects on diverse cell types within the intra-articular joint emerges as a promising avenue for future OA interventions. This paper reviews recent findings that underscore the significant role of tissue-specific exerkines in OA, delving into the underlying cellular and molecular mechanisms involved.

## 1 Introduction

Osteoarthritis (OA) is a prevalent and debilitating condition representing a steadily increasing and substantial health burden with deep implications for individuals, healthcare systems, and broader socioeconomic spheres. The cost of OA in the United States, Canada, United Kingdom, France and Australia has been estimated to account for between 1% and 2.5% of the gross national product of these countries (Hunter and Bierma-Zeinstra, 2019). Data from the Canadian cohort found that the average annual cost per OA patient was $12,200 (Hunter et al., 2014). Primary OA results from a combination of risk factors, such as genetics, dietary, estrogen, bone density, with increasing age and obesity being the most prominent (Yao et al., 2023). Globally, due to the aging global populations, the prevalence of OA has increased by 113.25%, from 247.51 million in 1990 to 527.81 million cases in 2019 (Long et al., 2022). Globally, an estimated 240 million individuals suffer from symptomatic mobility-restricted OA, with the knee being the most commonly affected joint and, thereby, the focal point of this review (Conaghan et al., 2019; Yao et al., 2023). Roughly 30% of individuals above the age of 45 exhibit radiographic indications of knee OA, half of whom experience symptoms associated with the disease (Katz et al., 2021). OA manifests through pathological alterations in cartilage, bone, synovium, tendons, muscles, and periarticular fat, engendering joint dysfunction, pain, stiffness, functional impairments, and the forfeiture of crucial activities (Abramoff and Caldera, 2020; Katz et al., 2021).

Current therapeutic approaches primarily encompass drug therapies such as non-steroidal anti-inflammatory drugs (NSAIDs) and joint replacement for advanced-stage disease; however, these strategies largely offer symptomatic relief (Latourte et al., 2020; Yue and Berman, 2022). In addition, NSAIDs have been shown to be associated with gastrointestinal, cardiovascular, renal, haematological and hepatic adverse events (AEs) (Holden et al., 2023). Joint replacement is only used for severe end-stage OA and imposes a heavy financial burden and pain on patients (Bandak et al., 2022). Recent clinical guidelines have increasingly advocated for non-surgical interventions such as physical exercise as frontline treatments (Hsu et al., 2023). Recognized as safe and efficacious, exercise therapy is gaining acceptance, with the World Health Organization recommending 150–300 min of moderate-intensity or 75–150 min of high-intensity physical activity weekly for early OA patients (Bennell et al., 2022; Chow et al., 2022). Over the past decade, a burgeoning body of evidence has affirmed the substantial impact of physical activity, notably moderate-intensity activity, on OA, corroborating the notion that “exercise is the real polypill,” predicated on organ-induced peripheral factors (Torstensen et al., 2023). Generally, the therapeutic effects of exercise on OA are attributed to repeated exercise sessions, suggesting an association with cumulative acute responses to physical activity.

Central to this discussion is the concept of “exerkines,” encompassing peptides, microRNAs, mRNAs, or other circulating RNA species released into the bloodstream in response to exercise (Lou et al., 2022; Robbins and Gerszten, 2023). This review delineates the potential function of exerkines in enhancing the benefits of exercise for OA patients. Exerkines involve various signaling entities secreted post-exercise, operating via endocrine, paracrine, and/or autocrine pathways (Chow et al., 2022). This analysis extends the exerkine classification to encompass exercise-associated humoral factors, including myokines (muscle), cardiokines (heart), and adipokines (white adipose tissue; WAT). Specially, only kines regulated by exercise can be called exerkines (Pillon et al., 2022; Hao et al., 2023). Recently, the pivotal role of physical exercise as a direct modulator enhancing general physiological aspects tied to OA has come to the fore, highlighting the exerkines’ function in this realm. Understanding the mechanistic variability in exercise responses and their mediating roles in OA exercise therapy is paramount, necessitating further exploration of the involved processes and mechanisms.

The novelty of this review compared to previous research is that we firstly reviewed the roles of exerkines in exercise therapy of OA. This review aims to encapsulate the current understanding of exerkines, underscoring their significance, delineating prevailing challenges (the specific molecular agents and mechanism remain unclear in the exercise therapy of OA), and envisioning prospective trajectories (exploring preclinical translational potentials, such as elucidating exerkine-associated effects and understanding individual physiological responses to different exercise interventions) in this burgeoning field.

## 2 Myokines

Skeletal muscle (SkM), the body’s largest organ, is closely associated with physical activity (Pedersen and Febbraio, 2008a). Recently, SkM has been recognized as a secreting organ that produces and releases cytokines called “myokines” (Pedersen and Febbraio, 2008a). These myokines—cytokines or peptides generated by skeletal muscle cells—find their way into the circulation, influencing other cells, tissues, or organs through autocrine, paracrine, or endocrine effects (Kurdiova et al., 2014; Pedersen, 2019; Severinsen and Pedersen, 2020). Several myokines, including irisin, interleukin-6 (IL-6), interleukin-15 (IL-15), meteorin-like (METRNL), and β-aminoisobutyric acid (BAIBA), are consistently released by SkM in response to exercise, playing a pivotal role in mediating the beneficial impacts of physical activity (Benatti and Pedersen, 2015; Paris et al., 2020).

### 2.1 Irisin

In 2012, Bostrom et al. (2012) uncovered that physical exercise stimulates muscles to secrete irisin, a hormone-like polypeptide derived from the cleavage of the fibronectin type III domain-containing protein 5 (FNDC5). Comprising 112 amino acid residues, irisin possesses a molecular weight of roughly 12 kD. Subsequent research established that exercising augments the expression of peroxisome proliferator-activated receptor-γ (PPAR-γ) and its coactivator-1-α (PGC-1α) in muscles, facilitating the downstream production of FNDC5 and its proteolytic conversion into irisin (Kim et al., 2018; Maak et al., 2021; Bao et al., 2022).

Vadala et al. (2020) were the first to demonstrate that irisin could potentially rejuvenate osteoarthritic chondrocytes, encouraging their proliferation while curtailing catabolism by deactivating p38, Akt, JNK, and NFκB *in vitro*, thus suggesting a cross-signaling mechanism between muscle and cartilage. Further, Wang et al. (2020) associated FNDC5 signaling disruption during OA knee development with increased chondrocyte apoptosis, whereas irisin was found to inhibit defective autophagy and enhance inflammatory chondrocyte and extracellular matrix (ECM) anabolism survival via the UCP-1 and Sirt3 signaling pathways. Li et al. (2021) noted that irisin could attenuate OA progression by reducing cartilage degradation and mitigating inflammation.

Jia et al. (2022) revealed that moderate-intensity treadmill exercise elevated irisin levels, exerting beneficial therapeutic effects on OA. These effects could be dampened using irisin-neutralizing antibodies. Moreover, irisin restored collagen II expression and reduced MMP-13 and ADAMTS-5 levels in IL-1β-induced OA chondrocytes by suppressing the PI3K/Akt/NF-κB signaling pathway and diminished pyroptosis in chondrocytes by inhibiting NLRP3/caspase-1 activity. The research concluded moderate mechanical stimulation could guard against chondrocyte pyroptosis through irisin-mediated PI3K/Akt/NF-κB pathway inhibition in osteoarthritis.

Further studies by He et al. (2020a) showed that irisin activated the ERK and p38 signaling pathways, with its anti-apoptotic effect reliant on ERK signaling, thus aiding in slowing OA progression by decreasing osteocyte apoptosis and enhancing subchondral bone microarchitecture. Additionally, irisin fostered chondrogenic differentiation in three-dimensional cultures of human articular chondrocytes (Posa et al., 2023). Cumulatively, these studies spotlight irisin’s promising role in augmenting bone density, resisting cartilage degradation, and maintaining joint environmental homeostasis through exercise training.

### 2.2 Interleukin-6 (IL-6)

During physical activity, contracting skeletal muscles extensively release interleukin-6 (IL-6) into the circulatory system (Pedersen and Febbraio, 2008b). Being a pleiotropic cytokine, IL-6 exhibits increased concentration in both the serum and synovial fluid of OA patients, establishing a correlation with radiographic knee OA (Hunter and Jones, 2015; Jones and Jenkins, 2018). Notably, about 50% of individuals with osteoarthritis endure synovitis, a significant contributor to IL-6 production in the condition (D’Agostino et al., 2005; Nguyen et al., 2017).

IL-6 operates through two principal pathways: classic signaling, facilitated by membrane-anchored IL-6R (mIL-6R), and trans-signaling, involving a soluble form of IL-6R (sIL-6R), which broadens the array of IL-6 target cells primarily guiding pro-inflammatory events (Kelly et al., 2021; Rodríguez-Hernández et al., 2022). The function of IL-6 in OA, however, remains a subject of dispute. Numerous investigations have linked heightened IL-6 levels in circulation to the prediction of osteoarthritis and cartilage deterioration, steering the potentiation of inflammatory repercussions in affected joints and promoting the creation of enzymes that break down chondrocyte ECM (Rose-John, 2012; Bergmann et al., 2017).

Yang et al. (2017) reported that DNA hypomethylation and histone hyperacetylation were observed in the IL-6 promoter regions in OA synovial fluid. Suzuki et al. (2010) reported that IL-6 induces synovial cells to produce MMP-1, MMP-3, and MMP-13. Nasi et al. (2016) found that basic calcium phosphate (BCP) crystal deposition increases in OA joints, stimulating IL-6 synthesis by articular chondrocytes. IL-6 stimulates IL-6 production in an autocrine manner and crystal deposition by inducing the calcification genes Ank, Anx5, and Pit1. This will result in the maintenance of BCP crystal-induced IL-6 production. This vicious circle induces cartilage matrix-degrading enzymes (such as Mmp-3 and Mmp-13 and Adamts-4 and Adamts-5) in chondrocytes, and subsequent chondrodegradation occurs.

However, some studies also showed that IL-6 levels were elevated in joints with symptomatic cartilage defects or osteoarthritis compared to healthy joints (Tsuchida et al., 2012). In addition, elevated IL-6 levels appear to promote the anabolism of resident chondrocytes and appear to facilitate the formation of new cartilage during *in vitro* regeneration (Tsuchida et al., 2012). Soluble IL-6R also augmented the production of anti-catabolic TIMPs in chondrocytes (Silacci et al., 1998), which suggests the direct role of IL-6 in regulating chondrocyte function and cartilage metabolism. This dual role of IL-6 is incompletely understood and may be caused by differential effects of IL-6 classic vs. trans-signaling (Wiegertjes et al., 2020).

### 2.3 Interleukin-7 (IL-7)

IL-7, a 25 kDa secreted globular protein encoded by the IL7 gene, has been discerned in the culture medium of human muscle tube primary cultures derived from satellite cells, with its concentration escalating over the cultivation period (Haugen et al., 2010). This molecule, called an “exerkine,” finds release into the bloodstream amid exercise (Andersson et al., 2010; Haugen et al., 2010).

Although it has been linked with osteoarthritis, the role of IL-7 in this context is mainly detrimental, contributing to the wreckage of cartilage in various joint disorders, including OA (Long et al., 2008; Zhang H. X. et al., 2016). Notably, IL-7 levels spike in the synovial fluid (SF) of older individuals with varying OA severities; however, it diminishes in cases of severe compartment 3 osteoarthritis, presumably owing to the substantial damage to cartilage cells enmeshed in the affected tissue (Long et al., 2008). Moreover, analysis in vervet monkeys indicated an elevated secretion of IL-7 in elderly meniscus samples with osteoarthritic changes compared to healthy counterparts, designating IL-7 as a marker rather than a therapeutic agent in OA management (Stone et al., 2015). The underlying consensus depicts IL-7 more as a marker for osteoarthritis rather than a therapeutic avenue leveraged through exercise.

### 2.4 Interleukin-15 (IL-15)

Interleukin-15 (IL-15), a glycoprotein comprising four intertwined α-helices and having a mass of 14–15 kDa (Steel et al., 2012), operates as an exercise-induced myokine, witnessing an uptick in its secretion from skeletal muscles post-exercise (Pedersen, 2011; Crane et al., 2015; Kurz et al., 2022). Early-stage knee OA patients have been observed to have elevated levels of IL-15 in the SF compared to those at the end stage (Scanzello et al., 2009). IL-15 seemingly aids in the recruitment or sustenance of CD8 lymphocytes within the joints of OA patients, hinting at its critical role in OA’s pathogenesis (Scanzello et al., 2009).

According to various studies, serum IL-15 (sIL-15) levels indicate the severity of early arthritis, and a surge in these levels is independently associated with heightened self-reported pain in knee OA sufferers (Gonzalez-Alvaro et al., 2011; Sun et al., 2013). Although identified as a target for pain management, IL-15 signaling may not significantly affect OA’s structural progression. Still, it does facilitate increased protease production in cartilage explants *in vitro* (Warner et al., 2020). It is regarded as a central regulator of MMPs, which spearhead cartilage loss in OA (Warner et al., 2020). Hence, targeting IL-15 signaling pathways could emerge as a pivotal strategy in OA therapeutic interventions, a premise necessitating more profound research.

### 2.5 Meteorin-like (METRNL)

Meteorin-like, or METRNL, is a circulating factor induced in muscles after exercise (Rao et al., 2014; Jung et al., 2018). Several evidence lines suggest anti-inflammatory and insulin-sensitizing effects of this myokine (Gao et al., 2022; Lu et al., 2022). In OA, Bridgewood et al. (2019) reported that METRNL is detectable in the SF of OA, which suggested that METRNL may participate in OA pathogenesis. Metrnl has also been reported to be expressed in hypertrophic chondrocytes. These hypertrophic chondrocytes have been reported as an essential event in the pathogenesis of osteoarthritis (Husa et al., 2010; Gong et al., 2016). The study also found that SF-metrnl was negatively associated with SF-MMP-13 and portrayed metrnl as a possible player in putative compensatory responses in OA (Sobieh et al., 2021). In other studies, they set an exercise protocol and discovered the correlation between the degree of OA and concentration of metrnl in the serum and synovial fluid. Moreover, based on the pathogenesis of OA, they found that exercise-induced metrnl could ameliorate OA through its anti-inflammatory and antipyroptotic effects, which are mediated by suppressing the PI3K/Akt/NFκB and NLRP3/caspase-1/GSDMD pathways (Liu et al., 2022).

### 2.6 Myostatin

Myostatin or growth differentiation factor 8 (GDF8) elevates with exercise. It exhibits a higher serum concentration in OA patients, correlating with disease severity (Kim et al., 2021; Omosule and Phillips, 2021). Pro-inflammatory cytokines such as TNF-α, IL-1, and IL-17 enhance myostatin expression in human synoviocytes. Moreover, chronic exposure to inflammatory cytokines leads to escalated myostatin expression in the synovium of hTNFtg mice (Dankbar et al., 2015). These observations propose a promising pathway for osteoarthritis treatment, wherein targeting myostatin could potentially reduce inflammation and joint destruction, paving the way for a new pharmacological approach in OA management.

### 2.7 Fractalkine

Fractalkine (FKN), also known as CX3CL1, is a novel membrane-bound myokine mainly expressed in contracting muscle (Altin and Schulze, 2011). Several studies show that aerobic exercise significantly elevates the fractalkine (Catoire et al., 2014; Crabb et al., 2016). Fractalkine is involved in various normal and pathological processes and is considered relevant to joint degeneration (Leonov et al., 2011; Wojdasiewicz et al., 2020). Fractalkine was significantly upregulated when chondrocytes underwent apoptosis, and blockage of the CX3CL1-CX3CR1 (CX3CL1 receptor) axis resulted in less bone resorption in OA (Guo et al., 2022). Fractalkine mediates cellular adhesive and migratory functions and is known to be expressed in mesenchymal stem cells destined to become chondrocytes (Feldman et al., 2013). While scant, available data from the literature confirm that CX3CL1 and its receptor are involved in OA. However, some opposite effects have been reported. Fractalkine has been detected in patients with osteoarthritis (OA) in SFs. The chemokine domain of FKN effectively induces the migration of OA fibroblasts (Klosowska et al., 2009). In addition, it was also reported that fractalkine activates c-Raf, MEK, ERK, and NF-κB on the MMP-3 promoter through CX3CR1, thus contributing to cartilage destruction during OA (Hou et al., 2017). Independent studies have paved the way for using CX3CL1 as a valuable marker for determining OA severity or monitoring treatment outcomes. However, assessing the occurrence and role of fractalkine and its receptor requires further, more detailed studies.

### 2.8 Lactate

Lactate is no longer considered a metabolic waste and a cause of muscle fatigue (Borchers and Pieler, 2010). Depending on the condition, such as during rest and exercise, following carbohydrate nutrition, injury, or pathology, lactate can serve as a myokine or exerkine with autocrine-, paracrine-, and endocrine-like functions that have essential basic and translational implications (Covington et al., 2016). Lactate is an exerkine produced during exercise in the integument and working muscles (Brooks et al., 2023). OA patient SF exhibited significantly increased levels of lactate secretion and aerobic glycolysis (Farah et al., 2022).

In OA, a concentration of 100 mM lactate with an exposure duration of 8 h is optimal for promoting chondrocyte ECM synthesis (Zhang X. et al., 2016), and pulsed lactate addition induced more Col2α1 expression (Zhang X. et al., 2016). On the other hand, the study also found that acidic pH caused by lactate exerts adverse effects on chondrocyte proliferation and ECM expression (Zhang X. et al., 2016). These observed differential biological effects of lactate on chondrocytes would have implications for the future design of polymeric cartilage scaffolds.

## 3 Cardiokines

The secretomes produced by the heart during exercise encompass a group of proteins that have been referred to as cardiokines. Recent findings reinforce the notion of the heart as a secretory organ that produces a variety of cardiac factors that can influence the function of various cell types. Cardiac factors may also participate in virial processes by acting on distal metabolic tissues and affecting systemic homeostasis. Cardiac factors have protective and harmful effects in osteoarthritis. In the pathological process, the imbalance produced by cardiac factors may lead to the result of disease.

### 3.1 Growth differentiation factor (GDF)-15

A part of the transforming growth factor-β superfamily, GDF-15 or macrophage-inhibitory cytokine 1 has been highlighted in recent studies for its increased circulation levels following exercise, both in mice and humans (Shimano et al., 2012; Klein et al., 2021; Wang et al., 2021). The cardiokine, besides exhibiting anti-inflammatory properties, is known to curtail oxidative stress, thereby signaling its potential as a therapeutic target in OA, a disease often preceded by inflammation and oxidation (Arnold et al., 2020; Kim et al., 2022; Zhang et al., 2022).

A recent study pinpointed GDF-15 as a central player inducing cellular senescence in OA-afflicted chondrocytes through MAPK14 activation, thereby suggesting its substantial role in advancing OA (Borchers and Pieler, 2010). Further research into GDF-15 could potentially unveil novel pathways to target in the treatment and management of OA.

### 3.2 Activin A

Activin A, a member of the transforming growth factor-β superfamily, shows a marked increase in response to acute exercise (Perakakis et al., 2018). Identified as an exercise-induced cardiokine, activin A has heightened concentrations in the SF of osteoarthritis (OA) patients. Furthermore, its production by synoviocytes and chondrocytes is stimulated in culture by inflammatory cytokines, including IL-1, TGF-beta, IFN-gamma, and IL-8 (Pignolo et al., 2019; Watt et al., 2020). Displaying immunomodulatory functions in OA, activin A governs the downregulation of type II collagen synthesis in articular cartilage at bone maturity and its reactivation during attempted OA repair, suggesting its role as a potential anabolic factor in cartilage (Hermansson et al., 2004; Diller et al., 2019).

### 3.3 Follistatin-like 1 (FSTL1)

FSTL1 originates from cardiomyocytes, endothelial cells, and smooth muscle cells (Xi et al., 2021). Exercise can enhance FSTL1 transcription in skeletal muscle and augment circulating FSTL1 levels (Görgens et al., 2013). Positioned as a potential biomarker of OA, FSTL1 can indicate the severity of joint damage, exhibiting a high presence at the juncture of eroding bone and inflammatory synovial pannus (Thornton et al., 2002; Mobasheri, 2012). Studies reveal FSTL1’s potential as a serum biomarker reflecting joint injury severity in OA patients (Wang et al., 2011; Elsadek et al., 2021). Specifically, it is a crucial pro-inflammatory factor in OA pathogenesis, fostering synoviocyte proliferation by activating the NF-κB pathway (Ni et al., 2015; Hu et al., 2019). Further research by Chaly et al. highlighted FSTL1’s regulatory role in chondrocyte activities and its implication in promoting apoptosis via the SAPK/JNK/Caspase3 pathway (Chaly et al., 2015; Xu et al., 2020). Cumulatively, these insights advocate targeting FSTL1 in osteoarthritis treatment strategies (Li et al., 2020).

### 3.4 Migration inhibitory factor (MIF)

MIF, a macrophage cytokine, modulates inflammatory and immune responses (Calandra and Roger, 2003). Produced by cardiomyocytes in the heart, its secretion amplifies during exercise, earning it the classification of a cardiokine (Moon et al., 2013; Chang et al., 2019). Observations show elevated MIF levels in the serum and SF of knee OA patients, establishing a strong correlation with the disease’s severity (Liu and Hu, 2012). Hence, MIF emerges as a potential new biomarker for evaluating knee OA risk and severity. Remarkably, MIF deletion can attenuate OA severity, with synovial fluid MIF levels independently associated with the severity of self-reported pain in OA patients (Zhang P. L. et al., 2016; Rowe et al., 2017).

### 3.5 Interleukin-33 (IL-33)

Primarily secreted by cardiac fibroblasts, IL-33 sees an uptick in expression and secretion in response to mechanical strain (Liew et al., 2016). Studies report a significant increase in IL-33 levels in OA chondrocytes, with double-stranded RNA promoting cartilage degeneration through the TLR3/IL-33 pathway (Li et al., 2017). These findings indicate that IL-33 chiefly originates from chondrocytes in an OA joint, not synovial fibroblasts, and plays a role in aggravating OA. Targeting IL-33 and enhancing IL-37 function may synergistically dampen inflammation, showcasing IL-33’s potential as a therapeutic target in OA management (Talabot-Ayer et al., 2012; Li et al., 2017; He et al., 2020b).

### 3.6 Secreted frizzled-related protein (SFRP)

SFRPs are glycoproteins encompassing a frizzled-like cysteine-rich domain, functioning as an exercise-inducible cardiokine (van Loon et al., 2021). Among the SFRPs, SFRP4 mRNA is highly responsive to exercise, potentially making it a crucial biomarker for evaluating the benefits of long-term exercise (Kim et al., 2017; Lee et al., 2019).

SFRPs regulate chondrocyte differentiation and survival in cartilage, presenting a viable target for therapeutic interventions (Loughlin, 2005). In a study utilizing a destabilization of the medial meniscus model (DMM), SFRP1 modulates joint homeostasis differentially in distinct joint compartments (Thysen et al., 2015). Diminished expression of SFRP1 renders the articular cartilage susceptible to premature aging and osteoarthritis (OA) development (Pasold et al., 2013). Conversely, SFRP3 protects chondrocytes in healthy articular cartilage by delaying the transformation of proliferative chondrocytes into hypertrophic ones (Snelling et al., 2007). In sum, SFRPs, including SFRP-1, -3, and -4, hold differential roles in OA pathogenesis.

### 3.7 Neuregulin

In human cardiac endothelial cells, neuregulin expression elevates in response to exercise, with neuregulin-4 (NRG4) playing a notable role as a member of the EGF-like family of extracellular ligands. Activated through ErbB4 receptor tyrosine kinases on the cytomembrane, NRG4 hinders OA progression by reducing inflammation, safeguarding chondrocytes from apoptosis, and mitigating ECM degradation via the MAPK/JNK signaling pathway (Cai et al., 2016; Yuan et al., 2023).

## 4 Adipokines

Originating from white adipose tissue, adipokines encompass bioactive peptides or proteins, immune molecules, and inflammatory mediators, including adiponectin and leptin (Neumann et al., 2016; Macdonald et al., 2019). Their incremented synthesis through exercise makes them focal points in OA exercise therapy.

### 4.1 Adiponectin

Adiponectin, predominantly secreted by adipocytes, sees heightened levels during exercise, implicating it in OA pathophysiology (Simpson and Singh, 2008; Bouassida et al., 2010; Martinez-Huenchullan et al., 2020). While increased severity of OA correlates with a decline in adiponectin levels in plasma and SF, its expression remains higher in OA patients compared to healthy individuals, associating it with OA prevalence (Honsawek and Chayanupatkul, 2010; Tang et al., 2018). Studies illustrate adiponectin’s protective role against OA by promoting chondrocyte proliferation and upregulating type II collagen and aggrecan in chondrocytes (Challa et al., 2010). Moreover, it inhibits the mitigation of OA chondrocyte calcification by activating the AMPK-mTOR signaling pathway, fostering autophagy (Duan et al., 2020). Interestingly, a favorable adiponectin/leptin ratio correlates with reduced pain in severe knee OA cases, spotlighting adiponectin’s potential as a surrogate biomarker for monitoring physical function in knee OA patients (Francin et al., 2014; Udomsinprasert et al., 2020).

### 4.2 Visfatin

Visfatin is a critical enzyme in essential cellular processes governed by NAD+, including aging, oxidative stress, and sirtuin signaling (Franco-Trepat et al., 2019a). This enzyme is notably increased following short-term moderate aerobic exercise, categorizing it as an exercise-induced adipokine (Seo et al., 2011; Plinta et al., 2012).

Visfatin has a pronounced role in the pathophysiology of osteoarthritis (OA), where it exhibits local effects on joints during the progression of the disease (Chen et al., 2010; Franco-Trepat et al., 2019b). Furthermore, visfatin elevates the expression of intercellular adhesion molecule type 1 (ICAM-1) in human OA synovial fibroblasts (OASFs), promoting the adherence of monocytes to OASFs and fostering angiogenesis through enhanced vascular endothelial growth factor (VEGF) expression (Law et al., 2020). It has also been associated with inducing apoptosis and oxidative stress in human OA chondrocytes (Cheleschi et al., 2018; Cheleschi et al., 2019).

From a biomechanical standpoint, visfatin disrupts microtubule and microfilament networks, influencing intracellular mechanics by decreasing intracellular elasticity and viscosity through glycogen synthase kinase 3β (GSK3β) inactivation (Chang et al., 2021). In summary, visfatin functions as a central catabolic agent in OA pathogenesis, mediating the deterioration of osteoarthritic cartilage (Yang et al., 2015).

### 4.3 Apelin

Apelin is a peptide ranging from 13 to 36 amino acids in length, whose biological functions are mediated through its specific receptor, APJ, a G-protein-coupled receptor with seven transmembrane domains (Vinel et al., 2018). Since its identification, apelin has been classified both as a myokine and an adipokine, and its levels are known to increase in response to exercise, categorizing it as an exerkine as well (Hehir and Morrison, 2012; Lee et al., 2022)].

In the context of OA, apelin is found in the SF, with its concentrations correlating positively with OA severity (Hu et al., 2011). This peptide aggravates OA pathogenesis through the apelin/APJ system, promoting chondrocyte proliferation and increasing the expression of catabolic factors such as MMP-1, -3, -9, and IL-1β. Moreover, it diminishes collagen II synthesis, highlighting its role as a catabolic factor in OA progression (Hu et al., 2010; Luo et al., 2021).

Research has indicated that reducing apelin expression can alleviate OA cartilage severity, presenting a potential therapeutic avenue (Chang et al., 2020). Apelin also augments IL-1β expression by activating the PI3K and ERK pathways in OASFs, underlining its contributive role in OA onset and/or progression (Chang et al., 2020). In light of these findings, apelin emerges as a significant factor in understanding the intricate pathophysiology of OA, offering a new perspective in OA research.

## 5 Discussion

**FIGURE 1:**
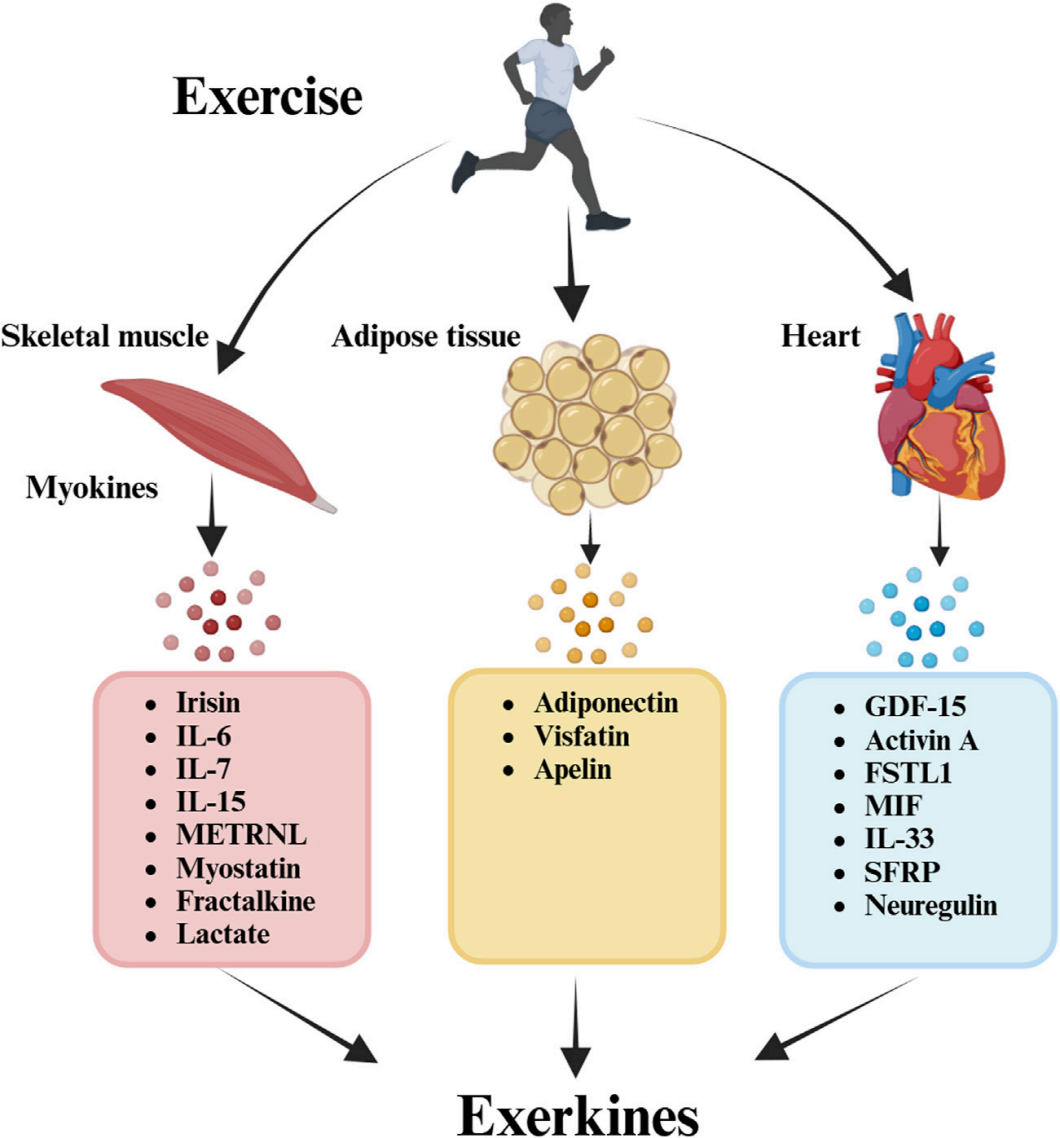
Exercise and exerkines. Exerkines involve various signaling entities secreted post-exercise, operating via endocrine, paracrine, and/or autocrine pathways. This analysis extends the exerkine classification to encompass exercise-associated humoral factors, including myokines (muscle), cardiokines (heart), and adipokines (white adipose tissue; WAT).

**FIGURE 2:**
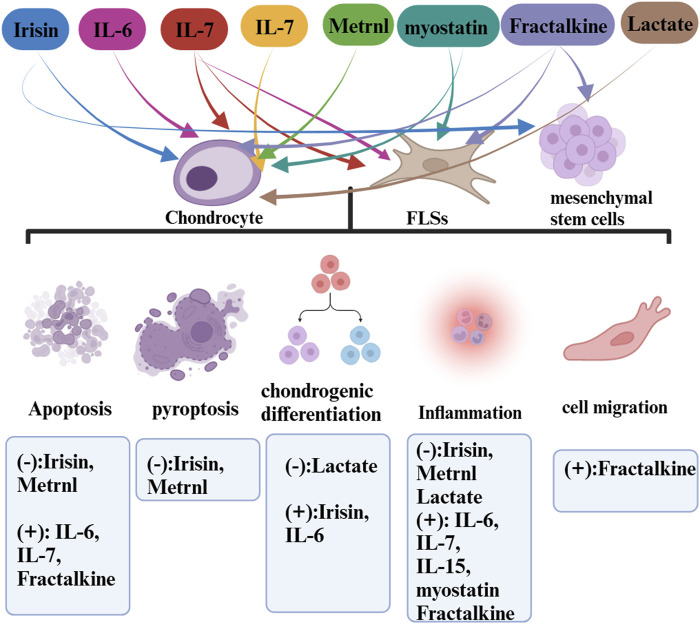
The roles of exerkines (myokines) in OA.

**FIGURE 3:**
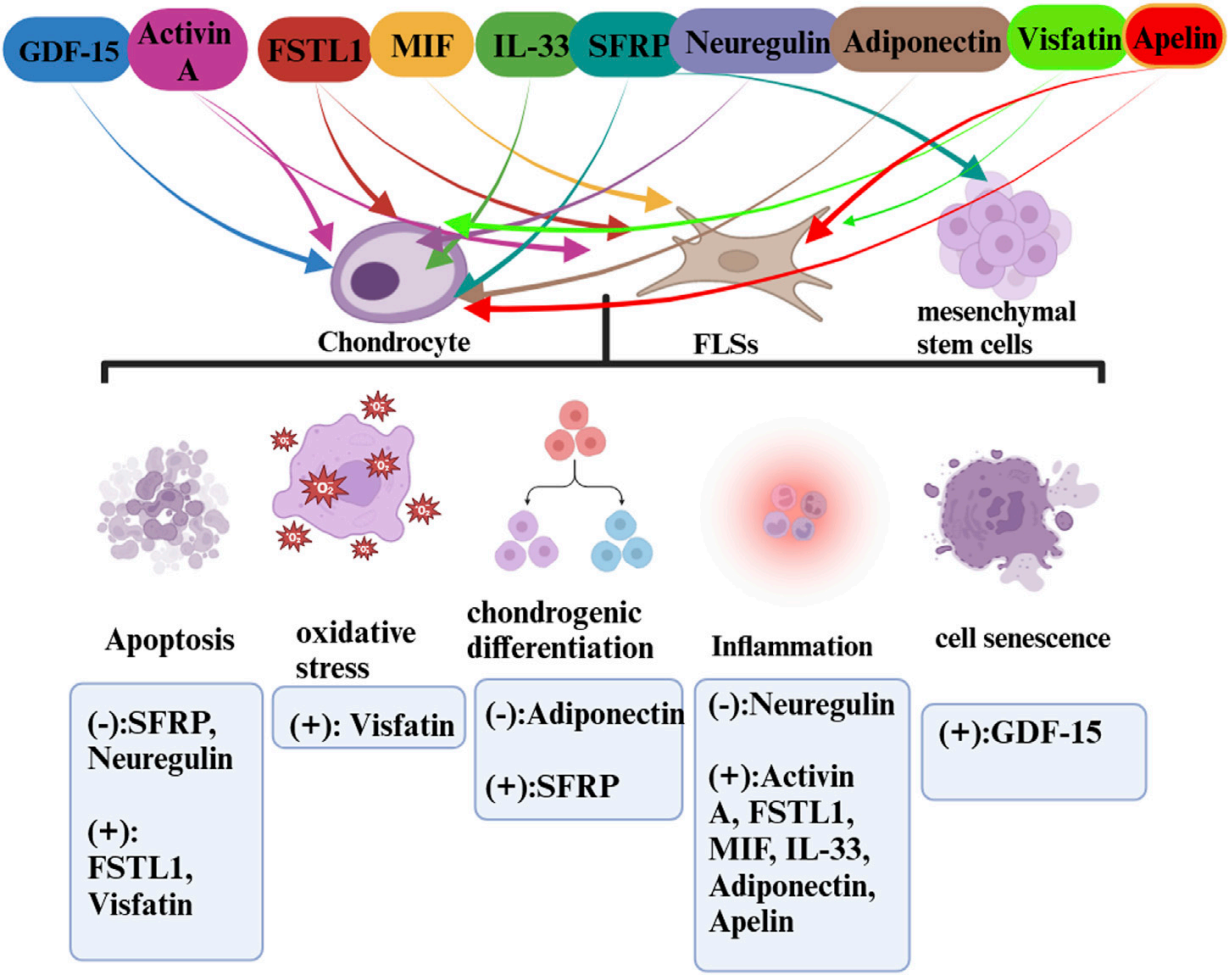
The roles of exerkines (cardiokines and adipokines) in OA.

Exercise is a pivotal facilitator of health benefits, fostering essential communication and coordination between various systems and organs [169]. While it is widely acknowledged that exercise is beneficial in mitigating the symptoms of osteoarthritis (OA), the underlying mechanisms and the extent of these benefits remain partially understood. The “exercise is medicine” paradigm stands to gain considerably by incorporating insights derived from exerkine research, thus opening new avenues in public health discourse (Figures 1–3).

OA is a heterogeneous and complicated disease which is characterized by articular cartilage damage, synovitis, subchondral bone remodeling and osteophyte formation (Holden et al., 2023). The susceptibility factors including systemic factors (genetics, aging, sex, race, physical labor, obesity, and hypertension) and local factors (abnormal joint strength lines, poor muscle strength, high-intensity exercise, and joint injury history) (Yao et al., 2023). The pathophysiological processes of OA involve multiple tissues and organs, and studying the communication between tissues is critical for exercise treatment of OA. In recent years, the skeletal muscle, heart, adipose tissue are considered secretory organs with endocrine functions that can produce and secrete exercise-related molecules which participate in exercise therapy of OA (Barbalho et al., 2020). Much of the existing research in this area has honed in on skeletal muscle as the principal source of exerkines. However, contemporary efforts are extending this focus to encompass other potential sources, including the heart and adipose tissues, to advance our understanding of how exercise aids in achieving health restoration and maintenance. Essentially, different bouts of exercise modulate the release of a variety of kines, namely, myokines, cardiokines, and adipokines, which were the focus of this review.

In detailing the influence of exercise-induced upregulation, we have excluded cytokines that are downregulated through an exercise from our discussion as they do not qualify as exerkines. Our analysis spans a range of myokines such as irisin, IL-6, IL-7, IL-15, METRNL, myostatin, fractalkine, and lactate, cardiokines like GDF-15, Activin A, FSTL1, MIF, IL-33, SFRP, and neuregulin, and adipokines such as adiponectin, visfatin, and apelin. Due to a scarcity of data, our discussion does not encompass the more recently identified exerkines and their potential roles in OA diseases. This review endeavors to bridge the gap between these disparate areas of study, presenting a consolidated view that hints at a larger, underlying theory. Firstly, exercise unleashes a complex network of endocrine interactions in which exerkines, released in response to exercise, interplay through inter-organ crosstalk and physiologic changes of OA. Secondly, the exerkines are released into the synovial fluid (SF) through the blood circulation. SF is formed by the serum in the articular capsule and released into the articular cavity, where it contributes to the unique functional properties of articular surfaces, provides nutrients to the cartilage, constitutes the microenvironment of articular cells, and modulates cells activity (Bolander et al., 2023). Thirdly, the exerkines act on FLSs, chondrocytes, mesenchymal stem cells and interfere with cell biological processes such as apoptosis, pyroptosis, inflammation, chondro differentiation through signaling pathway transduction to alleviate OA.

While the health benefits of exerkines are well acknowledged, it is also apparent that not all factors positively influence OA during exercise. Despite being upregulated through exercise, some factors can have adverse effects, a phenomenon possibly influenced by the intensity and frequency of exercise and the origin of the exerkines (as illustrated in Tables 1, 2). This discrepancy stems from the multifaceted impact of exercise on knee OA, involving elements such as mechanical stimulation and exerkine release, among others. We previously demonstrated that exercise could affect cartilage, which had a dual effect on osteoarthritis (Yang et al., 2019; Yang et al., 2020; Jia et al., 2022). Adaptive intensity exercise can reduce the sensitivity of chondrocytes to inflammation. However, excessive exercise leads to progressive damage, inhibits matrix synthesis, and stimulates the production of matrix degrading enzymes. Secondly, exercise can also affect articular cartilage by altering the surrounding microenvironment, such as exerkines (Yang et al., 2019; Yang et al., 2020; Jia et al., 2022; Liu et al., 2022). Research shows that normal articular cartilage and chondrocytes do not reap therapeutic benefits from mechanical stimulation alone; overstimulation can lead to damage [32, 170]. In circumstances where high-intensity exercises apply excessive mechanical stress to the articular cartilage, the detrimental effects can outweigh the potential benefits of exerkines. Even some exerkines have potential adverse effects in OA therapy as described in this review. Therefore, maintaining a harmonious balance between exercise and exerkines is critical.

**TABLE 1:** The roles of exerkines (myokines) in OA.

Exerkines	Origin	Effects in tissue	Immunomodulatory properties	Signaling pathway	Reference
Irisin (also known as FNDC5)	Myokine	Chondrocyte, mesenchymal stem cells	Positive correlation: anti-apoptosis; decreasing cartilage degradation and inhibiting inflammation and pyroptosis; chondrogenic differentiation	p38, Akt, JNK, NFκB, UCP-1, Sirt3,NLRP3, caspase-1, ERK	He et al., (2020a), Vadala et al., (2020), Wang et al., (2020), Li et al., (2021), Bao et al., (2022), Jia et al., (2022)
Interleukin-6	Myokine	Chondrocyte, FLSs	Positive correlation: promote the anabolism of chondrocytes; facilitate regeneration; production of anti-catabolic cytokines Negative correlation: increase synovitis; amplifies the inflammatory effects; DNA hypomethylation and histone hyperacetylation; produce MMPs; chondrodegradation	mIL-6R,sIL-6R, IL-6 trans-signaling	Silacci et al., (1998), Dagostino et al., (2005), Pedersen and Febbraio, (2008b), Andersson et al., (2010), Haugen et al., (2010), Suzuki et al., (2010), Rose-John, (2012), Tsuchida et al., (2012), Hunter and Jones, (2015), Nasi et al., (2016), Yang et al., (2017), Wiegertjes et al., (2020)
Interleukin-7	Myokine	Chondrocyte, FLSs	Negative correlation: cartilage destruction; increased in the SF of elderly with different degrees of OA	N/A	Pedersen, (2011), Steel et al., (2012), Crane et al., (2015), Stone et al., (2015)
Interleukin-15	Myokine	Chondrocyte	Negative correlation: recruitment or survival of CD8 lymphocytes; biomarker of disease severity; correlated with OA pain; increased protease and MMPs production	N/A	Rao et al., (2014), Jung et al., (2018), Warner et al., (2020), Gao et al., (2022), Lu et al., (2022)
Metrnl	Myokine	Chondrocyte, FLSs	Positive correlation: anti-inflammatory and antipyroptotic	PI3K/Akt/NFκB, NLRP3/caspase-1/GSDMD	Altin and Schulze (2011), Dankbar et al. (2015), Kim et al. (2021), Omosule and Phillips (2021), Liu et al. (2022)
Myostatin (also known as GDF8)	Myokine	Synoviocytes, chondrocyte	Negative correlation: correlate with the severity of OA and inflammatory cytokines	N/A	Leonov et al. (2011), Catoire et al. (2014), Crabb et al. (2016), Wojdasiewicz et al. (2020)
Fractalkine (also known as CX3CL1)	Myokine	Mesenchymal stem cells, chondrocyte, FLSs	Negative correlation: bone resorption; increase apoptosis; mediates cellular adhesive and migratory functions; migration of OA FLSs	CX3CR1, c-Raf, MEK, ERK, and NF-κB	Borchers and Pieler (2010), Zhang et al., (2016b), Covington et al., (2016), Hou et al., (2017), Farah et al., (2022), Brooks et al., 2023
Lactate	Myokine	Chondrocyte	Positive correlation: promoting chondrocyte ECM synthesis and Col2α1 expression	N/A	Klein et al., (2021), Kim et al., (2022)

**TABLE 2:** The roles of exerkines (cardiokines and adipokines) in OA.

Exerkines	Origin	Effects in tissue	Immunomodulatory properties	Signaling pathway	Reference
GDF-15	cardiokine	chondrocyte	Negative correlation: cellular senescence	MAPK14	Bennell et al. (2022)
Activin A	cardiokine	chondrocyte, FLSs	Positive correlation: anabolic factor in cartilage	mIL-6R, sIL-6R, IL-6 trans-signaling	Silacci et al. (1998), Dagostino et al. (2005), Pedersen and Febbraio (2008b), Andersson et al. (2010), Haugen et al. (2010), Suzuki et al. (2010), Rose-John (2012), Tsuchida et al. (2012), Hunter and Jones (2015), Nasi et al. (2016), Yang et al. (2017), Wiegertjes et al. (2020)
			Negative correlation: correlate with inflammatory cytokines		
FSTL1	Cardiokine	Chondrocyte, FLSs	Negative correlation: along the interface of eroding bone and inflammatory synovial pannus; pro-inflammatory in OA; FLSs proliferation; promote chondrocytes apoptosis	NF-κB pathway, SAPK/JNK/Caspase3	Calandra and Roger (2003), Liu and Hu (2012), Moon et al. (2013), Chaly et al. (2015), Ni et al. (2015), Chang et al., (2019), Hu et al. (2019), Li et al. (2020), Xu et al. (2020)
MIF	Cardiokine	FLSs	Negative correlation: correlate with the severity of OA and related pain	N/A	Talabot-Ayer et al. (2012), Li et al. (2017), He et al. (2020b)
Interleukin-33	Cardiokine	Chondrocyte	Negative correlation: increase inflammation and cartilage degradation	N/A	Loughlin (2005), Thysen et al. (2015), Kim et al. (2017), Lee et al. (2019)
Secreted frizzled-related protein	Cardiokine	Chondrocyte, mesenchymal stem cells	Positive correlation: regulate chondrocyte differentiation and survival; modulate joint homeostasis; delaying the maturation of proliferative chondrocytes into hypertrophic chondrocytes	polymorphism in SFRP cis-acting regulatory elements,	Bouassida et al. (2010), Neumann et al. (2016), Macdonald et al. (2019), Martinez-Huenchullan et al. (2020), Yuan et al. (2023)
Neuregulin	Cardiokine	Chondrocyte	Positive correlation: inhibiting the inflammation, protecting against apoptosis of chondrocyte, and decreasing the degradation of extracellular matrix	MAPK, JNK	Challa et al. (2010)
Adiponectin	Adipokines	Chondrocyte	Positive correlation: increase proliferation of chondrocytes and the type II collagen and aggrecan; alleviates the calcification of OA chondrocytes; matrix remodelling	AMPK, mTOR	Chen et al. (2010), Seo et al. (2011), Cheleschi et al. (2018), Franco-Trepat et al. (2019b), Cheleschi et al. (2019), Law et al. (2020), Chang et al. (2021)
Visfatin	Adipokines	Chondrocyte, FLSs	Negative correlation: enhances intercellular adhesion molecule type 1 expression; facilitates the adhesion of monocytes; induce apoptosis and oxidative stress; enhanced VEGF expression and facilitates angiogenesis; damages the microtubule and microfilament networks	GSK3β, ICAM-1, VEGF	Hu et al. (2010), Hehir and Morrison (2012), Yang et al. (2019), Barbalho et al. (2020), Chang et al. (2020), Yang et al. (2020), Luo et al. (2021), Bolander et al. (2023)
Apelin	adipokines	chondrocyte, FLSs	Negative correlation: exacerbated osteoarthritis pathogenesis; promoted chondrocyte proliferation and induced expression of MMPs and IL-1β	PI3K, ERK	Hu et al. (2010), Hu et al. (2011), Chang et al. (2020), Luo et al. (2021)

Future research endeavors must focus on exploring preclinical translational potentials, such as elucidating the clinical implications of exerkine-associated effects and understanding individual physiological responses to different exercise interventions. Given that the current body of exerkine research primarily consists of independent studies, there is a pressing need for research initiatives based on larger sample sizes and comprehensive panels to assess the cumulative impact of all exerkines, both at the joint level and in circulation. Finally, this review identifies gaps that remain in the field of exercise physiology science and opportunities that exist to translate biologic insights into osteoarthritis improvement.
